# Phylogenetic analysis of the genus *Taphrina*: proposal of one new genus and seven new species (*Taphrinales*, *Taphrinomycetes*) from China

**DOI:** 10.3897/imafungus.17.187200

**Published:** 2026-08-24

**Authors:** Chun-Yue Chai, Dan-Yang Cai, Bing-Yan Song, Rui-Xiu Wang, Feng-Li Hui

**Affiliations:** 1 School of Life Science, Nanyang Normal University, Nanyang 473061, China Research Center of Henan Provincial Agricultural Biomass Resource Engineering and Technology, Nanyang Normal University Nanyang China; 2 Research Center of Henan Provincial Agricultural Biomass Resource Engineering and Technology, Nanyang Normal University, Nanyang 473061, China School of Life Science, Nanyang Normal University Nanyang China

**Keywords:** *
Ascomycota
*, phylogenetic analysis, *
Taphrinomycotina
*, taxonomy

## Abstract

*Taphrina*, a genus within the family *Taphrinaceae* and the order *Taphrinales*, is a globally distributed group of plant-associated fungi. However, only three species of this genus have been previously reported in China. To enhance the understanding of species diversity within the *Taphrina* genus in China, sixteen yeast strains, isolated from leaf surfaces across various locations in China and initially identified as members of *Taphrina*, were examined from both phylogenetic and phenotypic perspectives. From these isolates, one new genus, *Srisukozyma*, is described to accommodate two new species, *Srisukozyma ovata* and *Sr. musae*, along with five additional new species *Taphrina aspidistrae*, *T. cunninghamiae*, *T. foliicola*, *T. fujianensis*, and *T. guizhouensis* which we describe. The descriptions of these taxa are supplemented by illustrations and results from phylogenetic analyses. Additionally, phenotypic comparisons with related genus and species are provided. This study contributes to the broader understanding of ascomycetous yeast diversity in natural environments and sets the stage for future taxonomic and ecological investigations.

## Introduction

*Taphrina*, the type genus of the family *Taphrinaceae*, was established by Fries in 1832 with *T. populina* as the type species (Fonseca and Rodrigues 2011). Initially, the genus was described as a sexual ascomycete fungus within the “*Archiascomycetes*” (Nishida and Sugiyama 1994), later being placed in the class *Taphrinomycetes*, which was formally proposed for archiascomycete lineages by Eriksson and Winka (1997). The asexual state of *Taphrina* was originally classified under the genus *Lalaria* (Eriksson and Winka 1997; Inácio et al. 2004). However, molecular analyses have unequivocally placed previously recognized *Lalaria* anamorphs within the monophyletic genus *Taphrina*, which belongs to the order *Taphrinales* (Sjamsuridzal et al. 1997; Rodrigues and Fonseca 2003; Fonseca and Rodrigues 2011). Therefore, Selbmann et al. (2014) emended the genus *Taphrina* and, in accordance with the International Code of Nomenclature for Algae, Fungi, and Plants (McNeill et al. 2012), proposed that each asexual morph (previously placed in *Lalaria*) be combined with its corresponding sexual morph into a single species within the genus *Taphrina*.

Most species of the genus *Taphrina* exhibit a dimorphic lifestyle. The sexual morph is filamentous, strictly biotrophic, and forms asci within the infected plant tissues (Rodrigues and Fonseca 2003; Fonseca and Rodrigues 2011; Petrýdesová et al. 2013). In contrast, the asexual morph is yeast-like, saprobic, reproduces by budding, and is easily cultured on artificial media (Tsai et al. 2014; Petrýdesová et al. 2016). Cultures are often pigmented, appearing cream, yellowish, or pinkish-cream in color, with pigmentation intensifying as they age (Selbmann et al. 2014). Notably, cultures of many *Taphrina* species are composed of yeast forms that have been isolated from infected tissues of the respective host plants using the spore-fall method (Fonseca and Rodrigues 2011).

Members of the genus *Taphrina* are parasitic pathogens of various vascular plants, including economically important fruit trees, shrubs, herbs, and some ferns (Mix 1949; Cissé et al. 2013; Tsai et al. 2014). Specific examples include *T. populina* on poplars (*Populus* spp.) (Robinson et al. 2011; Tsai et al. 2014), *T. ulmi* on elms (*Ulmus* spp.) (Bond 1956), *T. wiesneri* on flowering cherry (*Prunus yedoensis*) (Tsai et al. 2014; Lee et al. 2025), and *T. caerulescens* on oaks (*Quercus* spp.) (Sutton and Hodges 1990). *T. deformans* is the most extensively studied species in the genus due to its significance as a plant pathogen worldwide, with all known peach and nectarine cultivars being susceptible to infection (Cissé et al. 2013; Oh et al. 2020). One of the most notable symptomatic effects is the severe distortion and reddening of the leaves (Kern and Naef-Roth 1975). These symptoms are typically linked to the production of indole-3-acetic acid (IAA) by *T. deformans* (Yamada et al. 1990; Tsai et al. 2014). Another significant species is *T. flavorubra*, the causative agent of deformed fruits, or plum pocket, in plums (Rodrigues and Fonseca 2003; Cissé et al. 2013). Infected fruits exhibit abnormal enlargement, deformation, and a spongy texture. Additionally, *T. betulina* significantly reduces the mean height and diameter of infected *Betula pubescens* (birch trees) (Spanos and Woodward 1994), potentially leading to losses in timber production, especially in short-rotation forestry (Selbmann et al. 2014).

*Taphrina* is an ancient, species-rich ascomycete genus, with nearly 100 scientific names recorded under it (Wang et al. 2020). Unfortunately, approximately 70% of the species described have not been deposited in culture collections. Rodrigues and Fonseca (2003) clarified the generic boundaries of *Taphrina* using a morpho-molecular approach. Due to low DNA sequence divergence and the absence of available strains, the number of accepted *Taphrina* species was reduced to 28 (Fonseca and Rodrigues 2011). Since then, nine additional species have been recognized based on phenotypic and phylogenetic analyses (Petrýdesová et al. 2013, 2016; Selbmann et al. 2014). Currently, 38 valid species are accepted in the genus *Taphrina* (Petrýdesová et al. 2016). However, only four *Taphrina* species—*T. cerasi*, *T. deformans*, *T. mume*, and *T. pruni*—have been reported in China (Wang et al. 2022). In the present study, strains of *Taphrina*-like taxa were isolated from different locations in China. To clarify their taxonomic status, both phylogenetic and phenotypic analyses were conducted. Five new species are described within *Taphrina*, and one new genus, accommodating two new species, is introduced within the *Taphrinomycetes*.

## Materials and methods

### Sample collection and yeast isolation

Fresh leaves were collected from Fujian, Guizhou, Hainan, and Henan Provinces of China. Yeast strains were isolated from the leaf surfaces using an improved spore-fall method, as described by Nakase and Takashima (1993). In brief, the leaves were cut into small pieces and attached to the inner lid of a Petri dish using a thin layer of petroleum jelly. The dish contained yeast extract-malt extract (YM) agar (0.3% yeast extract, 0.3% malt extract, 0.5% peptone, 1% glucose, and 2% agar), with 0.01% chloramphenicol added to prevent bacterial growth. The plates were incubated at 20 °C and monitored daily for colony formation. Colonies were selected and purified by streaking onto separate YM agar plates. After purification, yeast strains were suspended in YM broth containing 20% (v/v) glycerol and stored at −80 °C.

### Phenotypic examination

Phenotypic characteristics were determined following the standard methods described in Kurtzman et al. (2011). Colony and cell morphology: Colony morphology was examined after 7 days of incubation at 20 °C on YM agar. Cell morphology was observed using a LEICA DM2500 microscope (LEICA, Wetzlar, Germany) after 3 days of growth in YM broth at 20 °C, and analyzed with LASV4.13 software. Glucose fermentation: Fermentation was tested using Durham fermentation tubes incubated at 20 °C for 14 days; gas production indicated a positive reaction. Carbon assimilation: Tests were performed in liquid medium containing 0.67% yeast nitrogen base (YNB) and 0.5% of the test carbon compound. Incubation was at 20 °C for 28 days; growth (turbidity) was recorded as positive. Nitrogen assimilation: Starved inoculum was prepared by growing yeasts in YNB broth for 48 h, followed by centrifugation, two washes with sterile distilled water, and resuspension in sterile water. The test medium consisted of 1.77% yeast carbon base (YCB) and the specified concentration of the test nitrogen compound. After 28 days of incubation at 20 °C, growth was evaluated by turbidity. Pseudohyphae formation: The Dalmau plate method on corn meal agar (CMA: 2.5% corn meal infusion and 2% agar) was used, with sterile coverslips creating an anaerobic environment. Plates were incubated at 20 °C for 14 days and examined microscopically. Sexual reproduction: Single strains or strain pairs were inoculated on CMA, 5% malt extract (ME) agar (5% malt extract and 1.5% agar), Fowell’s acetate agar (0.5% sodium acetate and 2% agar), Gorodkowa agar (0.1% glucose, 0.5% sodium chloride, 1% peptone, and 2% agar), and V8 agar (10% V8 juice and 2% agar), and incubated at 20 °C for up to two months; asci and ascospores were examined periodically. Additional growth tests: Growth at different temperatures (15, 20, 25, 30, 35, and 37 °C) was assessed on YM agar. Growth in vitamin-free medium, tolerance to 1% acetic acid, 0.01% cycloheximide, and 10% NaCl plus 5% glucose, starch-like polysaccharide production (Lugol’s iodine), urease activity (Christensen’s urea agar), and Diazonium Blue B (DBB) reaction were all performed according to the standard protocols in Kurtzman et al. (2011). All novel taxonomic descriptions and proposed names were deposited in the MycoBank database (http://www.mycobank.org; accessed 10 December 2025).

### Molecular sequencing

Genomic DNA was extracted from yeast strains using the Ezup Column Yeast Genomic DNA Purification Kit following the manufacturer’s instructions (Sangon Biotech, Co., Ltd., Shanghai, China; Cat. No. B518225-0100). The primer pairs ITS5 (5’-GGAAGTAAAAGTCGTAACAAGG-3’)/ITS4 (5’-TCCTCCGCTTATTGATATGC-3’) (White et al. 1990), NL1 (5’-GCATATCAATAAGCGGAGGAAAAG-3’)/NL4 (Kurtzman and Robnett 1998), and SSU1 (5’-CTGGTTGATCCTGCCAGTAG-3’) /SSU5 (5’-TGATCCTTCTGCAGGTTCACCTAC-3’) (Petrýdesová et al. 2013) were selected to amplify the internal transcribed spacer (ITS) region, the D1/D2 domain of the large subunit (LSU) rRNA gene, and the partial mitochondrial small rRNA subunit (*mtSSU*) region, respectively. PCR amplification was carried out in a 25 µL reaction volume containing 9.5 µL of ddH_2_O, 12.5 µL of 2 × Taq PCR Master Mix with blue dye (Sangon Biotech Co., Shanghai, China), 1 µL of DNA template, and 1 µL of each primer. The PCR protocols were as follows: for the ITS region, an initial denaturation at 95 °C for 3 min, followed by 34 cycles at 94 °C for 40 s, 57.2 °C for 45 s, and 72 °C for 1 min, with a final extension at 72 °C for 10 min; for the LSU region, an initial denaturation at 94 °C for 1 min, followed by 34 cycles at 94 °C for 30 s, 55.2 °C for 1 min, and 72 °C for 1.5 min, with a final extension at 72 °C for 10 min (Chai et al. 2023); for the *mtSSU* region, an initial denaturation at 94 °C for 3 min, followed by 36 cycles at 94 °C for 45 s, 50 °C for 2 min, and 72 °C for 2 min, with a final extension at 72 °C for 5 min (Petrýdesová et al. 2013). The PCR products were sequenced using the same primers at Sangon Biotech Co., Ltd., Shanghai, China. Forward and reverse sequences were manually edited using BioEdit v7.1.3.0 (Hall 1999) by removing primer sequences, trimming low-quality ends based on chromatogram quality scores (Phred < 20), and resolving ambiguous base calls manually. The edited forward and reverse reads were then assembled into consensus sequences using the same software. Sequence similarity searches were performed using the BLASTN 2.2.19 algorithm (Zhang et al. 2000) against the GenBank database. All newly generated sequences were deposited in GenBank (https://www.ncbi.nlm.nih.gov/genbank/).

### Phylogenetic analyses

Phylogenetic analyses were based on combined sequences of the ITS, LSU, and *mtSSU* regions. Sequences from various *Taphrinomycotina* species retrieved from GenBank were combined with those generated in our study. Information on the studied yeasts and their corresponding GenBank accession numbers is provided in Table 1. Sequences for each locus were aligned using MAFFT v.7.110 (Katoh and Standley 2013) with the G-INI-I option (Katoh et al. 2005). The resulting alignments were visualized using MEGA v.11 (Tamura et al. 2021). To avoid inclusion of poorly aligned or highly gapped positions, the alignments were manually trimmed by removing ambiguous regions at both ends as well as any positions with >90% gaps or unresolved bases; all trimming was performed under visual inspection within MEGA v.11. Phylosuite v.1.2.2 (Zhang et al. 2020) was used to concatenate the trimmed alignments from the different loci. The best-fit evolutionary models for the alignments were estimated using jModelTest (Guindon and Gascuel 2003; Posada 2008) under the Akaike information criterion.

**Table 1.** The accession numbers of studied yeasts and reference sequences retrieved from the GenBank database.

**Taxa name**	**Strain no**.	**Substrate or host**	**Location**	**GenBank accession no**.			**References**
				**ITS**	**LSU D1/D2**	***mtSSU***	
*Novakomyces olei*	NCAIM Y.02187^T^	Olive oil	Spain	NR_172188	MG250349	–	Čadež et al. 2021
*Protomyces arabidopsidicola*	DSM 110145^T^	*Arabidopsis thaliana* (Leaf)	Finland	LT602858	MK934482	–	Wang et al. 2021
*Protomyces inouyei*	NRRL YB-4354^T^	*Crepis* sp. (Pedicel)	Japan	NR_121206	NG_042406	KU134844	Petrýdesová et al. 2016
*Protomyces inundatus*	NRRL Y-6349^T^	–	England	MK937057	U76528	–	Kurtzman and Robnett 1998
*Protomyces gravidus*	NRRL Y-17093^T^	*Bidens cernua*	USA	MK937055	NG_064601	–	Kurtzman and Robnett 1998
*Protomyces lactucaedebilis*	NRRL YB-4353 ^T^	*Lactuca debilis* (Leaf)	Japan	MK937058	U84343	–	Kurtzman and Robnett 1998
*Protomyces macrosporus*	NRRL Y-12879^T^	–	USA	MK937059	U94939	–	Kurtzman and Robnett 1998
*Protomyces pachydermus*	NRRL YB-4355^T^	*Taraxacum officinale*	England	MK937060	NG_064602	–	Kurtzman and Robnett 1998; Petrýdesová et al. 2016
*Saitoella coloradoensis*	NRRL YB-2330^T^	Insect frass	USA	NR_137778	NG_059942	JN161163	Kurtzman and Robnett 2012; Petrýdesová et al. 2016
*Saitoella complicata*	NRRL Y-17804^T^	Soil	Bhutan	NR_111655	NG_027621	KU134846	Goto et al. 1987; Petrýdesová et al. 2016
*Savitreella phatthalungensis*	PYCC 9005^T^	*Ananas comosus* (Leaf)	Thailand	MW879743	MW876306	–	Nutaratat et al. 2022
***Srisukozyma musae***	**NYNU 24437** ^T^	***Musa nana***	**China**	**PP849688**	**PP837701**	**PX939036**	**This study**
***Srisukozyma musae***	**NYNU 244126**	***Musa nana***	**China**	**PX070522**	**PX070523**	**PX939145**	**This study**
***Srisukozyma musae***	**NYNU 244111**	***Musa nana***	**China**	**PX070525**	**PX070524**	**PX939143**	**This study**
***Srisukozyma musae***	**NYNU 24449**	***Musa nana***	**China**	**PX070527**	**PX070526**	**PX939037**	**This study**
***Srisukozyma ovata***	**NYNU 2411186^T^**	***Polygonum chinense***	**China**	**PV843161**	**PV839685**	**PX939146**	**This study**
***Srisukozyma ovata***	**NYNU 2411221**	***Polygonum chinense***	**China**	**PX070537**	**PX070536**	**PX939149**	**This study**
*Taphrina alni*	CBS 683.93^T^	*Alnus incana*	Austria	AF492077	AF492024	KU134812	Rodrigues and Fonseca 2003; Petrýdesová et al. 2016
*Taphrina americana*	CBS 331.55^T^	*Betula fontinalis*	USA	NR_155874	AF492025	KU134813	Rodrigues and Fonseca 2003; Petrýdesová et al. 2016
*Taphrina antarctica*	CCFEE 5198^T^	Sandstone	Antarctica	NR_132870	NG_059477	–	Selbmann et al. 2014
*Taphrina arrabidae*	PYCC 5735^T^	*Pistacia lentiscus*	Portugal	PX671092	NG_059909	–	Inácio et al. 2004
***Taphrina aspidistrae***	**NYNU 2411134^T^**	***Aspidistra elatior***	**China**	**PV839642**	**PV839641**	**PX939148**	**This study**
*Taphrina aspidistrae*	JS-42	–	Korea	JF706660	JF706659	–	Direct from Genbank
*Taphrina betulina*	CBS 119536^T^	*Betula ‘intermedia’*	Sweden	AF492080	AF492027	KU134847	Rodrigues and Fonseca 2003; Petrýdesová et al. 2016
*Taphrina bullata*	CBS 1278 ^T^	*Pyrus communis*	Slovakia	KC491200	JN997390	KC494909	Petrýdesová et al. 2013; Petrýdesová et al. 2016
*Taphrina caerulescens*	CBS 351.35^T^	*Quercus marylandica*	USA	NR_155875	NG_057691	KU134815	Rodrigues and Fonseca 2003; Petrýdesová et al. 2016
*Taphrina carpini*	CBS 102169^T^	*Quercus pyrenaica*	Slovakia	NR_119488	NG_042399	KU134814	Inácio et al. 2004; Petrýdesová et al. 2016
*Taphrina communis*	CBS 352.35^T^	*Prunus americana* (Leaf and Fruit)	USA	NR_160070	NG_070567	KU134816	Rodrigues and Fonseca 2003; Petrýdesová et al. 2016
*Taphrina confusa*	CBS 375.39^T^	*Prunus virginiana* (Flower and Fruit)	USA	NR_155876	AF492035	–	Rodrigues and Fonseca 2003
***Taphrina cunninghamiae***	**NYNU 232134^T^**	***Cunninghamia lanceolata***	**China**	**OQ851904**	**OQ851903**	**PX939040**	**This study**
***Taphrina cunninghamiae***	**NYNU 232156**	***Cunninghamia lanceolata***	**China**	**PX070522**	**PX070523**	**PX939039**	**This study**
*Taphrina dearnessii*	CBS 334.55^T^	*Acer rubrum* (Leaf)	USA	NR_172284	AF492037	–	Rodrigues and Fonseca 2003; Petrýdesová et al. 2016
*Taphrina deformans*	CBS 356.35^T^	*Prunus persica*	Netherlands	NR_160071	MH867217	KU134817	Rodrigues and Fonseca 2003; Petrýdesová et al. 2016
*Taphrina epiphylla*	CBS 111109^T^	*Alnus incana*	Slovakia	AF492096	AF492039	KU134818	Rodrigues and Fonseca 2003; Petrýdesová et al. 2016
*Taphrina flavorubra*	CBS 377.39^T^	*Prunus susquehanae* (Fruit)	USA	NR_160073	MH867541	KU134819	Rodrigues and Fonseca 2003; Petrýdesová et al. 2016
***Taphrina foliicola***	**NYNU 2211137^T^**	***Carpinus turczaninowii***	**China**	**OP959653**	**OP959502**	**PX939147**	**This study**
***Taphrina foliicola***	**NYNU 221115**	***Carpinus turczaninowii***	**China**	**OP954718**	**OP954686**	**PX939038**	**This study**
***Taphrina foliicola***	**NYNU 236334**	***Cornus officinalis***	**China**	**PX063790**	**PX070528**	**PX939043**	**This study**
***Taphrina foliicola***	**NYNU 236258**	***Carpinus turczaninowii***	**China**	**PX063794**	**PX070529**	**PX939042**	**This study**
*Taphrina foliicola*	CGMCC 2.2537	–	China	PQ309310	PQ309490	–	Direct from Genbank
***Taphrina fujianensis***	**NYNU 224115^T^**	***Dicranopteris* sp**.	**China**	**OP278686**	**OP278685**	**PX939041**	**This study**
*Taphrina fujianensis*	CGMCC 2.5682	–	China	PQ309311	PQ309491	–	Direct from Genbank
***Taphrina guizhouensis***	**NYNU 24380^T^**	***Cunninghamia lanceolata***	**China**	**PV839639**	**PV839638**	**PX939035**	**This study**
***Taphrina guizhouensis***	**NYNU 243137**	***Rhododendron simsii***	**China**	**PX070534**	**PX070535**	**PX939144**	**This study**
*Taphrina gei-montani*	CBS 14159^T^	*Geum montanum*	Slovakia	KU134800	–	KU134828	Petrýdesová et al. 2016
*Taphrina inositophila*	PYCC 5733^T^	*Acer monspessulanum*	Portugal	NR_111147	AY239199	–	Inácio et al. 2004
*Taphrina insititiae*	CBS 12782^T^	*Prunus insititia*	Slovakia	KC491202	JN997391	KC494911	Petrýdesová et al. 2013; Petrýdesová et al. 2016
*Taphrina johansonii*	CBS 378.39^T^	*Populus tremuloides*	USA	KC511055	NG_057692	KC494916	Rodrigues and Fonseca 2003; Petrýdesová et al. 2016
*Taphrina kurtzmanii*	PYCC 5737^T^	*Cistus albidus*	Portugal	PX671093	NG_059910	–	Inácio et al. 2004
*Taphrina letifera*	CBS 335.55^T^	*Acer spicatum* (Leaf)	USA	NR_155877	NG_057693	KU134820	Rodrigues and Fonseca 2003; Petrýdesová et al. 2016
*Taphrina mirabilis*	CBS 357.35^T^	*Prunus angustifolia*	USA	NR_155878	NG_070568	–	Rodrigues and Fonseca 2003
*Taphrina padi*	CBS 693.93^T^	*Prunus padus* (Fruit)	Germany	NR_155879	AF492048	KC494912	Rodrigues and Fonseca 2003; Petrýdesová et al. 2016
*Taphrina populina*	CBS 337.55^T^	*Populus nigra* (Leaf)	Sweden	NR_145337	NG_057694	KU134821	Rodrigues and Fonseca 2003; Petrýdesová et al. 2016
*Taphrina populi-salicis*	CBS 419.54^T^	*Populus trichocarpa* (Leaf)	USA	NR_155881	NG_057695	KU134822	Rodrigues and Fonseca 2003; Petrýdesová et al. 2016
*Taphrina pruni*	CBS 119537^T^	*Prunus domestica*	Slovakia	AF492111	AF492056	KU134823	Rodrigues and Fonseca 2003; Petrýdesová et al. 2016
*Taphrina pruni-subcordatae*	CBS 381.39^T^	*Prunus subcordata*	Slovakia	NR_155880	NG_070569	–	Rodrigues and Fonseca 2003
*Taphrina rhizophora*	CBS 1278^T^	*Populus alba*	Slovakia	KC491201	KC491203	KC494917	Petrýdesová et al. 2013; Petrýdesová et al. 2016
*Taphrina robinsoniana*	CBS 382.39^T^	*Alnus incana*	Slovakia	NR_172369	NG_057696	KU134824	Rodrigues and Fonseca 2003; Petrýdesová et al. 2016
*Taphrina sacchari*	CBS 119738^T^	*Acer saccharum*	USA	AF492117	AF492061	–	Rodrigues and Fonseca 2003
*Taphrina sadebeckii*	CBS 102170^T^	*Alnus glutinosa*	Germany	NR_155882	NG_057697	KU134825	Rodrigues and Fonseca 2003; Petrýdesová et al. 2016
*Taphrina tormentillae*	CBS 339.55^T^	*Potentilla canadensis*	USA	NR_155883	NG_057698	KU134845	Rodrigues and Fonseca 2003; Petrýdesová et al. 2016
*Taphrina tosquinetii*	CBS 276.28^T^	*Alnus glutinosa* (Leaf)	Europe	NR_155884	NG_057699	KU134826	Rodrigues and Fonseca 2003; Petrýdesová et al. 2016
*Taphrina ulmi*	CBS 420.54^T^	*Ulmus rubra* (Leaf)	USA	NR_155885	NG_057700	KU134827	Rodrigues and Fonseca 2003
*Taphrina veronaerambellii*	PYCC 5734^T^	*Acer monspessulanum*	Portugal	NR_111148	NG_059911	NG_059911	Inácio et al. 2004; Petrýdesová et al. 2016
*Taphrina vestergrenii*	CBS 679.93^T^	*Dryopteris clix-mas*	Europe	NR_155886	NG_057701	–	Rodrigues and Fonseca 2003
*Taphrina virginica*	CBS 340.55^T^	*Ostrya virginiana*	USA	AF492125	AF492071	–	Inácio et al. 2004
*Taphrina viridis*	CCY 58-9-1^T^	*Alnus alnobetula*	Slovakia	PQ013128	PQ013143	PQ013155	Petrýdesová et al. 2016
*Taphrina wiesneri*	CBS 275.28^T^	*Prunus avium*	–	NR_160065	AF492072	KC494915	Rodrigues and Fonseca 2003
*Schizosaccharomyces cryophilus*	CBS 11777^T^	*Osmia rufa* (Feed)	Denmark	NR_121468	GU470882	NC_040930	Kurtzman and Robnett 1998
*Schizosaccharomyces japonicus*	CBS 354^T^	Strawberry wine	Japan	NR_121199	U94943	–	Kurtzman and Robnett 1998
*Schizosaccharomyces osmophilus*	CBS 15793^T^	*Osmia florisomnis* (Bee bread)	Germany	MK589403	MK253005	NC_071386	Kurtzman and Robnett 1998
*Schizosaccharomyces pombe*	CBS 356^T^	Beer	Africa	MK749863	NG_042649	AF442355	Kurtzman and Robnett 1998, 2003
*Saccharomyces cerevisiae*	CBS 1171^T^	–	France	AB018043	JQ689017	KX657745	Kurtzman and Robnett 1998; Sulo et al. 2017

Note. ^T^, type strain. Species obtained in this study are in bold.

Maximum Likelihood (ML) and Bayesian Inference (BI) analyses were performed using RAxML v.8.2.3 (Stamatakis 2014) and MrBayes v.3.2.7a (Ronquist et al. 2012), respectively. For the ML analysis, a GTRGAMMA model of evolution was applied, and bootstrap (BS) values were assessed through 1,000 rapid bootstrap replicates. In the BI analysis, six Markov Chain Monte Carlo (MCMC) chains were run simultaneously for 50 million generations under the best-fit evolutionary models selected using jModelTest (Guindon and Gascuel 2003; Posada 2008), with trees sampled every 1,000 generations. The first 25% of the generated trees were discarded as burn-in, and the remaining trees were used to estimate Bayesian posterior probabilities (BPPs) for the clades. Each tree was visualized with BS values and BPPs in Figtree v.1.4.3 (Andrew 2016).

## Results

### Molecular phylogeny

A total of 73 sequences, including the outgroup *Saccharomyces cerevisiae* CBS 1171, were used in the phylogenetic analyses to determine the taxonomic placement of the isolates obtained in this study. The concatenated dataset of the three regions across the 73 samples had a total length of 1,979 bp, consisting of 598 bp for the ITS region, 574 bp for the LSU region, and 807 bp for the *mtSSU* region. The phylogeny generated from the combined ITS, LSU, and *mtSSU* dataset strongly supported *Taphrinales* as an independent order (BS = 100%, BPPs = 1.0). Within *Taphrinales*, five new lineages emerged from *Taphrina*, and two new independent clades clustered with *Savitreella phatthalungensis*, with full support.

**Figure 1.**
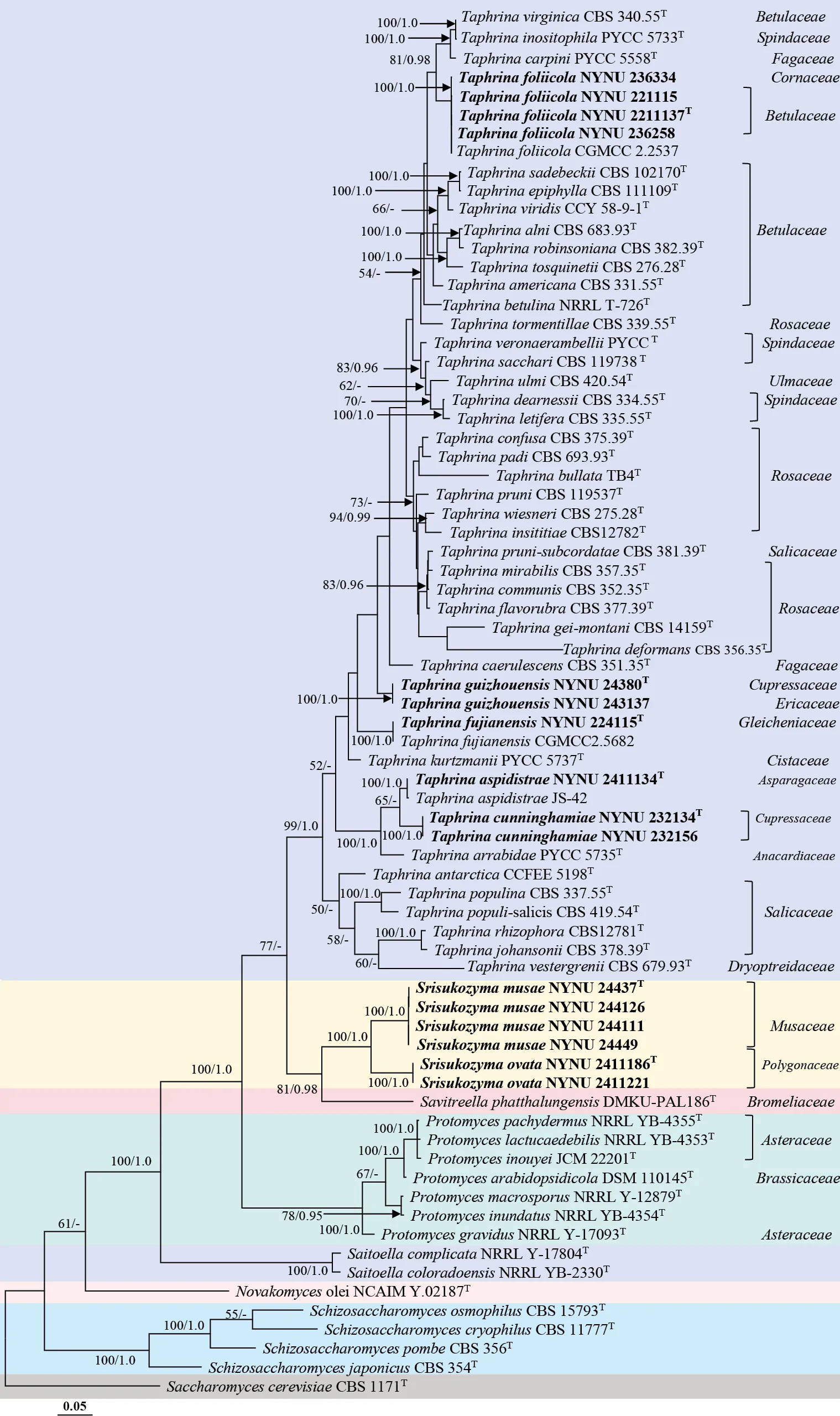
Phylogenetic positions of the newly studied strains within *Taphrinomycotina*, inferred from the combined ITS, LSU, and *mtSSU* dataset. The tree topology was generated using the maximum likelihood method, with bootstrap values and Bayesian posterior probabilities (above 50% and 0.95, respectively) indicated at the nodes. The tree was rooted with *Saccharomyces cerevisiae* CBS 1171. Sequences from type strains are marked with a (T), and the newly proposed species are highlighted in bold.

Ten strains, NYNU 2211137, NYNU 221115, NYNU 236334, NYNU 236258, NYNU 224115, NYNU 24380, NYNU 243137, NYNU 2411134, NYNU 232134, and NYNU 232156, formed five groups within the genus *Taphrina*, represented by NYNU 2211137, NYNU 224115, NYNU 24380, NYNU 2411134, and NYNU 232134 (Fig. 1). Group NYNU 2211137, containing four strains with one nucleotide substitution in the ITS region, had identical LSU sequences with strain CGMCC 2.2537 and two nucleotide ITS sequences difference, which indicated that they were conspecific. This group formed a clade clustered with *T. carpini*, *T. inositophila*, and *T. virginica* with significant support (Fig. 1) and differed from those three known species in this clade by more than three nucleotide substitutions (0.5%) in the LSU region and over 35 nucleotide mismatches (5.9%) in the ITS region. Based on these results, we conclude that the group NYNU 2211137 corresponds to a novel species, for which we propose the name *Taphrina foliicola* sp. nov.

Strain NYNU 224115 clustered with strain CGMCC 2.5682, sharing identical LSU sequences and similar ITS sequences (with two nucleotide differences), which indicated that they belong to same species. Group NYNU 24380, consisting of two strains with identical sequences, indicating that they are also conspecific. These two groups formed two separate branches within the genus *Taphrina* (Fig. 1). They differed from each other by nine nucleotide substitutions (1.6%) and 40 nucleotide mismatches (6.3–6.6%) in the LSU and ITS regions, respectively. They also differed from other *Taphrina* species by more than nine nucleotide substitutions (1.6%) in the LSU region and more than 57 nucleotide substitutions (9.1%) in the ITS region. Based on these differences, we conclude that these two groups represent two novel *Taphrina* species. Therefore, we propose *Taphrina fujianensis* sp. nov. and *Taphrina guizhouensis* sp. nov. to accommodate the groups NYNU 224115 and NYNU 24380, respectively.

Strain NYNU 2411134 clustered with strain JS-42, sharing identical ITS sequences and similar LSU sequences (with a three nucleotides difference), suggesting that they are the same species. Groups NYNU 232134, including two strains with identical sequences, and 2411134 were closely related to *T. arrabidae* (Fig. 1). These two groups differed from *T. arrabidae* by 14 nucleotide substitutions (2.4%) in the LSU region and by greater than 38 nucleotide mismatches (6.1%) in the ITS region. Groups NYNU 232134 and 2411134 showed 15 nucleotide substitutions (2.6%) in the LSU region and more than 9% nucleotide mismatches in the ITS region. Based on these findings, we propose two new species, *Taphrina aspidistrae* sp. nov. and *Taphrina cunninghamiae* sp. nov., to accommodate the groups NYNU 2411134 and NYNU 232134.

Six strains, NYNU 24437, NYNU 244126, NYNU 244111, NYNU 24449, NYNU 2411186, and NYNU 2411221, formed two distinct branches closely related to *Savitreella* (Fig. 1), a new genus recently described by Nutaratat et al. (2022). The group represented by NYNU 24437 differed from the group represented by NYNU 2411186 by 33 nucleotide substitutions (5.7%) in the LSU region and by 85–88 nucleotide mismatches (13.4–13.9%) in the ITS region. A BLASTn search of the LSU sequences revealed that the two groups showed 88–90% similarity to genera within the *Taphrinales*, such as *Taphrina*, *Protomyces*, and *Savitreella*. However, only 25–35% of the ITS sequence of these groups matched taxa from these genera when using ITS sequences as the query. The taxonomic thresholds predicted by Vu et al. (2016) to discriminate current yeast genera were 97.11% for LSU and 96.31% for ITS. Given the low sequence similarity to any known taxa and strong phylogenetic support within *Taphrinales*, it is appropriate to establish one new genus *Srisukozyma* within *Taphrinales*. Therefore, we propose *Srisukozyma musae* sp. nov. and *Srisukozyma ovata* sp. nov. for the groups NYNU 24437 and NYNU 2411186, respectively.

### Taxonomy

#### Taphrina aspidistrae

Taxon classificationFungiHaplotaxidaTaphrinaceae

C.Y. Chai & F.L. Hui sp. nov.

EAF3CCC7-3F36-5478-A863-274704D9DEF7

861665

Fig. 2A, B

##### Etymology.

The specific epithet aspidistrae refers to *Aspidistra*, the plant genus from which the type strain was isolated.

**Figure 2.**
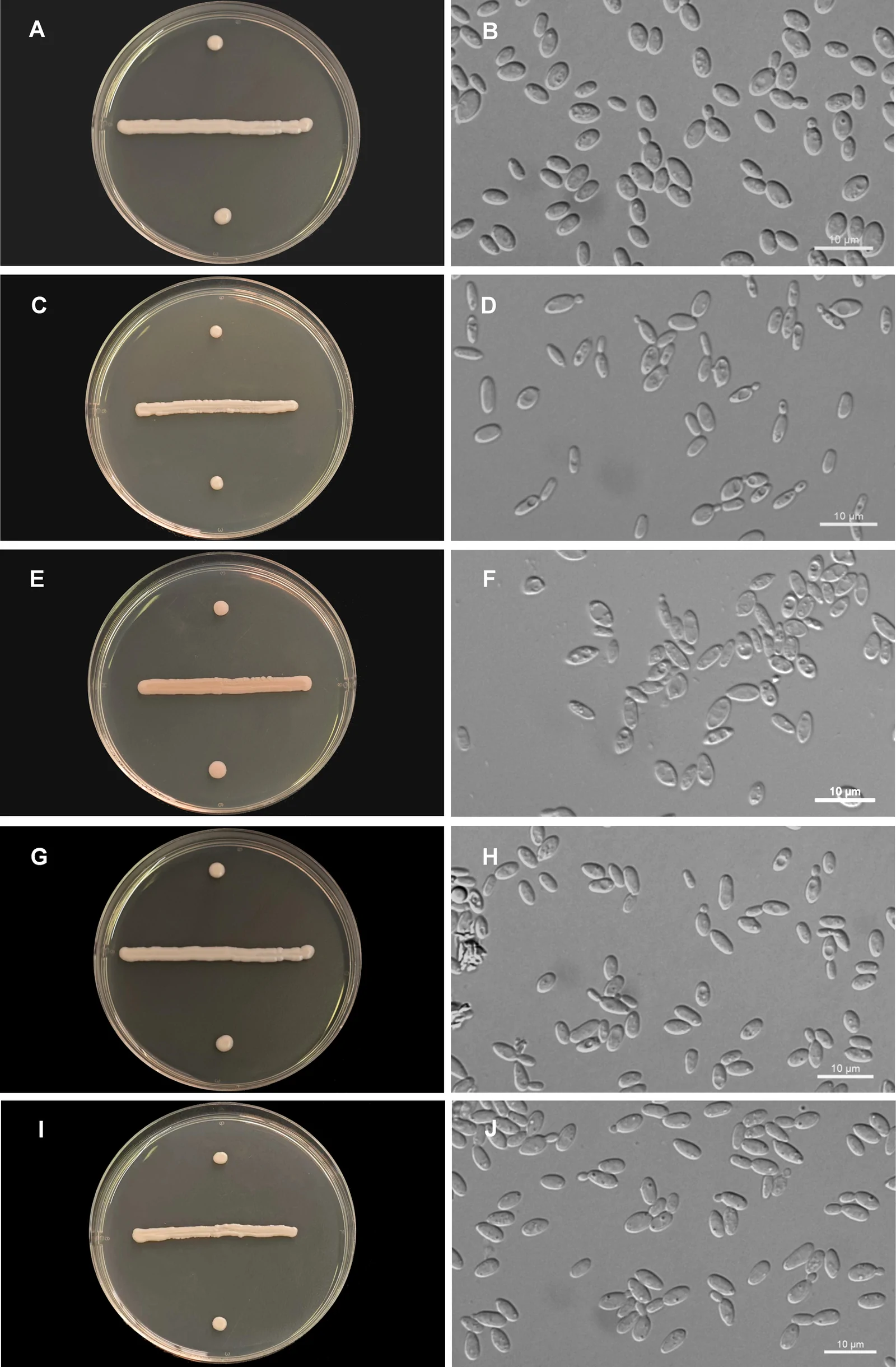
The streak culture grown on YM agar for 7 d at 20 °C and vegetative cells grown in YM broth for 3 d at 20 °C. **A, B**. *T. aspidistrae* NYNU 2411134. **C, D**. *T. cunninghamiae* NYNU 232134. **E, F**. *T. fujianensis* NYNU 224115. **G, H**. *T. guizhouensis* NYNU 24380. **I, J**. *T. foliicola* NYNU 2211137. Scale bars: 10 μm.

##### Diagnosis.

In the multi-gene phylogenetic analysis, *T. aspidistrae* clustered as sister to *T. cunninghamiae* and *T. arrabidae* with high support values (Fig. 1). *T. aspidistrae* differed from the closely related species *T. cunninghamiae* and *T. arrabidae* by 14–15 nucleotide substitutions (2.4–2.6%) in the LSU region and by 40–57 nucleotide mismatches (6.4–9%) in the ITS region (Suppl. material 2: table SS1). Physiologically, *T. aspidistrae* can be distinguished from *T. cunninghamiae* in its inability to assimilate trehalose, maltose, ethanol, and DL-lactate. It can also be distinguished from *T. arrabidae* in its ability to assimilate inulin, methyl-α-D-glucoside, L-arabinose, D-ribose, ribitol, and D-glucitol as well as grow in vitamin-free medium (Table 2; Suppl. material 2: table SS1).

**Table 2.** Physiological and biochemical characteristics that differ between the new species and closely related species.

**Characteristics**	**1**	2*	3*	4*	**5**	**6**	7*	8*	**9**	10*	**11**	**12**	**13**	14*
Carbon assimilation														
Inulin	d	w	+	–	+	+	–	–	+	–	+	–	+	N
Sucrose	+	+	+	+	+	+	–	–	+	+	+	+	+	+
Raffinose	d/w	–	+	+	–	–	–	–	–	–	–	+	d	–
Trehalose	+	+	+	+	+	w	s	+	–	–	+	+	+	+/w
Maltose	+	+	+	+	+	d	–	–	–	–	+	d/w	+	+
Melezitose	+	+	+	+	+	w	–	–	w	+	+	+	+	+
Methyl-α-D-glucoside	–	–	+	d/–	+	d	–	–	d/w	–	+	–	d/w	–
Cellobiose	+	+	+	+	+	d	s	+	w	+	+	–	–	S
Salicin	d/w	+	+	d	+	w	–	+	w	S	+	d/w	d/w	W
L-Sorbose	–	–	–	–/d	–	–	–	v	–	–	–	d	–	–
L-Rhamnose	–	–	–	–	–	–	–	–	–	–	+	–	–	–
D-Xylose	+	+	+	v	+	w	s	+	+	+	+	+	d	+/s
L-Arabinose	–	+	+	v	+	d/w	–	–	d/w	–	+	–	d/w	–
D-Arabinose	–	w	+	+	+	+	–	–	+	–	+	–	dw	–
D-Ribose	–	w	+	v	+	d/w	–	–	d	–	+	–	–	–
Ethanol	–	–	+	v	–	–	+	–	–	–	w	w	+	N
Ribitol	–	w	w	d	+	+	–	v	d/w	–	+	d/w	d/w	N
D-Mannitol	+	+	+	+	+	w	s	+	w	+	+	+	+	+/w
D-Glucitol	+	+	+	+	w	d	+	–/s	d	–	+	d/w	d	–
DL-Lactate	–	w	–	–	–	–	–	–	–	–	+	+	d/w	+/s
Succinate	d	+	+	+	–	+	+	+	+	+	+	+	+	+
Citrate	–	+	+	+	d	–	+	+	d/w	+	+	–	+	–
D-Gluconate	–	+	–	d/–	–	–	–	–	–	–	–	–	–	+
Growth tests														
Growth in vitamin-free medium	w	w	w	–/+	+	+	–	v	+	–	+	+	+	–
Growth at 30 °C	–	–	–	–	+	–	–	–	–	–	–	+	+	+
Growth at 35 °C	–	–	–	–	–	–	–	–	–	–	–	–	–	+

Note. 1, *T. foliicola*; 2, *T. carpini*; 3, *T. virginica*; 4, *T. inositophila*; 5, *T. fujianensis*; 6, *T. guizhouensis*; 7, *T. kurtzmanii*; 8, *T. caerulescens*; 9, *T. aspidistrae*; 10, *T. arrabidae*; 11, *T. cunninghamiae*; 12, *Sr. musae*; 13, *Sr. ovata*; 14, *Sa. phatthalungensis*. +, positive reaction; –, negative reaction; d, delayed positive; n, not available; s, slowly positive; v, variable; w, weakly positive. All data from this study, except* which were obtained from the original description (Fonseca and Rodrigues 2011; Inácio et al. 2004; Nutaratat et al. 2022).

##### Typus.

CHINA • Guizhou Prov.: Guiyang City, Guiyang Botanical Garden, on the phylloplane of *Aspidistra elatior*, Oct. 2024, D. Lu, NYNU 2411134 (holotype GDMCC 2.541^T^ preserved as a metabolically inactive state, culture ex-type JCM 38266).

##### Description.

On YM agar after 7 days at 20 °C, the streak culture is yellowish-cream, butyrous, and smooth, with an entire margin. In YM broth after 3 days at 20 °C, the cells are ellipsoidal and cylindrical, 2.3–3.6 × 3.8–9.4 μm, occurring singly or with buds. After 1 month at 20 °C, a pellicle and a sediment are present. In Dalmau plate culture on CMA, simple pseudohyphae are formed. Ascospores formation is not observed on CMA, 5% ME agar, Fowell’s acetate agar, Gorodkowa agar or V8 agar. Fermentation is absent. The following carbon compounds are assimilated in liquid medium: glucose, inulin, sucrose, melezitose (weak), methyl-α-D-glucoside (delayed and weak), cellobiose (weak), salicin (weak), D-xylose, L-arabinose (delayed and weak), D-arabinose, D-ribose (delayed), glycerol, ribitol (delayed and weak), D-mannitol (weak), D-glucitol (delayed), succinate, and citrate (delayed and weak). No growth occurred in raffinose, melibiose, galactose, lactose, trehalose, maltose, L-sorbose, L-rhamnose, 5-keto-D-gluconate, methanol, ethanol, erythritol, galactitol, myo-inositol, DL-lactate, D-gluconate, D-glucosamine, N-acetyl-D-glucosamine, 2-keto-D-gluconate, D-glucuronate, and glucono-1,5-lactone. Nitrate, nitrite (weak), and ethylamine (delayed and weak) are utilized as a nitrogen source. L-Lysine and cadaverine are not utilized. Growth in vitamin-free medium is positive. No growth occurred with 1% acetic acid, 0.01% cycloheximide or 10% NaCl plus 5% glucose. Maximum growth temperature is 25 °C. Starch-like polysaccharide is produced. Urease reaction is positive. Diazonium blue B reaction is negative.

##### GenBank accession numbers.

Holotype GDMCC 2.541^T^ (ITS: PV839642, D1/D2: PV839641, *mtSSU*: PX939148).

##### Notes.

An unpublished strain, JS-42 isolated from Korea, had identical ITS sequences and similar LSU sequences (with a three nucleotides difference) with *T. aspidistrae*, which indicated that this new species may be also found in other locations than China.

#### Taphrina cunninghamiae

Taxon classificationFungiHaplotaxidaTaphrinaceae

C.Y. Chai & F.L. Hui sp. nov.

5C5DA1AF-F406-5E8E-BF05-4F7DAA702B35

861666

Fig. 2C, D

##### Etymology.

The specific epithet cunninghamiae refers to *Cunninghamia*, the plant genus from which the type strain was isolated.

##### Diagnosis.

Multi-gene phylogenetic analysis revealed that *T. cunninghamiae* formed a distinct lineage closely related to *T. arrabidae* and *T. aspidistrae* (Fig. 1). *T. cunninghamiae* differed from *T. arrabidae* and *T. aspidistrae* by 14–15 nucleotide substitutions (2.4–2.6%) in the LSU region and 57–67 nucleotide mismatches (9–10.6%) in the ITS region. Physiologically, *T. cunninghamiae* can be distinguished from *T. arrabidae* and *T. aspidistrae* in its ability to assimilate trehalose, maltose, L-rhamnose, ethanol, and DL-lactate (Table 2; Suppl. material 2: table SS2).

##### Typus.

CHINA • Guizhou Prov.: Pingtang County, Sifangjing Village, on the phylloplane of *Cunninghamia lanceolata*, Feb. 2023, D. Lu, NYNU 232134 (holotype GDMCC 2.492^T^ preserved as a metabolically inactive state, culture ex-type PYCC 9978).

##### Description.

On YM agar after 7 days at 20 °C, the streak culture is yellowish-cream, butyrous, and smooth, with an entire margin. In YM broth after 3 days at 20 °C, the cells are ellipsoidal and cylindrical, 2.2–4.4 × 4.3–6.6 μm, occurring singly or with buds. After 1 month at 20 °C, a pellicle and a sediment are present. In Dalmau plate culture on CMA, pseudohyphae are not formed. Ascospores formation is not observed in any of the strains or when strains are paired on CMA, 5% ME agar, Fowell’s acetate agar, Gorodkowa agar or V8 agar. Fermentation is absent. The following carbon compounds are assimilated in liquid medium: glucose, inulin, sucrose, trehalose, maltose, melezitose, methyl-α-D-glucoside, cellobiose, salicin, L-rhamnose, D-xylose, L-arabinose, D-arabinose, D-ribose, ethanol (weak), glycerol, ribitol, D-mannitol, D-glucitol, DL-lactate, succinate, citrate, and N-acetyl-D-glucosamine (weak). No growth occurred in raffinose, melibiose, galactose, lactose, L-sorbose, 5-keto-D-gluconate, methanol, erythritol, galactitol, myo-inositol, D-gluconate, D-glucosamine, 2-keto-D-gluconate, D-glucuronate, and glucono-1,5-lactone. Nitrite, nitrate, and L-lysine are utilized as a nitrogen source. Ethylamine and cadaverine are not utilized. Growth in vitamin-free medium is positive. No growth occurred with 0.01% cycloheximide or 10% NaCl plus 5% glucose. Maximum growth temperature is 25 °C. Starch-like polysaccharide is produced. Urease reaction is positive. Diazonium blue B reaction is negative.

##### Additional strain examined.

CHINA • Guizhou Prov.: Pingtang County, Sifangjing Village, on the phylloplane of *Cunninghamia lanceolata*, Feb. 2023, D. Lu, NYNU 232156.

##### GenBank accession numbers.

Holotype GDMCC 2.492^T^ (ITS: OQ851904, D1/D2: OQ851903, *mtSSU*: PX939040); additional strains NYNU 232156 (ITS: PX070522, D1/D2: PX070523, *mtSSU*: PX939039).

#### Taphrina fujianensis

Taxon classificationFungiHaplotaxidaTaphrinaceae

C.Y. Chai & F.L. Hui sp. nov.

58C4FC54-7C00-5B72-82E9-6C0908FFBC6B

861667

Fig. 2E, F

##### Etymology.

The specific epithet fujianensis refers to the geographic origin of the type strain, Quanzhou city, Fujian Province.

##### Diagnosis.

Multi-gene phylogenetic tree showed that *T. fujianensis* formed a distinct lineage and was sister to *T. guizhouensis* (Fig. 1). *T. fujianensis* differed from *T. guizhouensis* by nine nucleotide substitutions (1.6%) and 40 nucleotide mismatches (6.6%) in the LSU and ITS regions, respectively. Physiologically, *T. fujianensis* can be differentiated from *T. guizhouensis* in its inability to assimilate succinate and its ability to assimilate citrate and grow at 30 °C (Table 2; Suppl. material 2: table S3).

##### Typus.

CHINA • Fujian Prov.: Quanzhou City, Qingyuan Mountain, on the phylloplane of *Dicranopteris* sp., Mar. 2022, W.T. Hu, NYNU 224115 (holotype GDMCC 2.310^T^ preserved as a metabolically inactive state, culture ex-type PYCC 9943).

##### Description.

On YM agar after 7 days at 20 °C, the streak culture is pinkish-cream, butyrous, and smooth, with an entire margin. In YM broth after 3 days at 20 °C, the cells are ovoid, ellipsoidal and cylindrical, 1.9–3.1 × 3.6–5.9 μm, occurring singly or with buds. After 1 month at 20 °C, a pellicle and a sediment are present. In Dalmau plate culture on CMA, pseudohyphae and hyphae are not formed. Ascospores formation is not observed on CMA, 5% ME agar, Fowell’s acetate agar, Gorodkowa agar or V8 agar. Fermentation is absent. The following carbon compounds are assimilated in liquid medium: glucose, inulin, sucrose, trehalose, maltose, melezitose, methyl-α-D-glucoside, cellobiose, salicin, L-rhamnose (weak), D-xylose, L-arabinose, D-arabinose, D-ribose, glycerol, ribitol, D-mannitol, D-glucitol (weak), citrate (delayed), and N-acetyl-D-glucosamine (weak). No growth occurred in raffinose, melibiose, galactose, lactose, L-sorbose, 5-keto-D-gluconate, methanol, ethanol, erythritol, galactitol, myo-inositol, DL-lactate, succinate, D-gluconate, D-glucosamine, 2-keto-D-gluconate, D-glucuronate, and glucono-1,5-lactone. Nitrite, nitrate (weak), and L-lysine are utilized as a nitrogen source. Ethylamine and cadaverine are not utilized. Growth in vitamin-free medium is positive. No growth occurred with 1% acetic acid, 0.01% cycloheximide or 10% NaCl plus 5% glucose. Maximum growth temperature is 30 °C. Starch-like polysaccharide is produced. Urease reaction is positive. Diazonium blue B reaction is negative.

##### GenBank accession numbers.

Holotype GDMCC 2.310^T^ (ITS: OP278686, D1/D2: OP278685, *mtSSU*: PX939041).

##### Notes.

Another Chinese strain CGMCC 2.5682 shared an identical LSU sequence and a similar ITS sequence (with only two nucleotides differences) with *T. fujianensis*, indicating that this new species has a wide geographical distribution.

#### Taphrina guizhouensis

Taxon classificationFungiHaplotaxidaTaphrinaceae

C.Y. Chai & F.L. Hui sp. nov.

E83B5BA5-F229-5E09-A0CE-54971B0D55FC

861668

Fig. 2G, H

##### Etymology.

The specific epithet guizhouensis refers to the geographic origin of the type strain, Pingtang County, Guizhou Province.

##### Diagnosis.

The new species *T. guizhouensis* formed a distinct lineage, closely related to *T. fujianensis* (Fig. 1). *T. guizhouensis* differed from *T. fujianensis* by more than nine nucleotide substitutions (1.6%) and 40 nucleotide mismatches (6.6%) in the LSU and ITS regions, respectively (Suppl. material 2: table S4). Physiologically, *T. guizhouensis* can be distinguished from *T. fujianensis* in its ability to assimilate succinate and its inability to assimilate citrate and grow at 30 °C (Table 2; Suppl. material 2: table S4).

##### Typus.

CHINA • Guizhou Prov.: Pingtang County, on the phylloplane of *Cunninghamia lanceolata*, Mar. 2024, D. Lu, NYNU 24380 (holotype GDMCC 2.543^T^ preserved as a metabolically inactive state, culture ex-type JCM 38268).

##### Description.

On YM agar after 7 days at 20 °C, the streak culture is yellowish-cream, butyrous, and smooth, with an entire margin. In YM broth after 3 days at 20 °C, the cells are ovoid, ellipsoidal and cylindrical, 1.7–3.1 × 3.5–8.8 μm, occurring singly or with buds. After 1 month at 20 °C, a pellicle and a sediment are present. In Dalmau plate culture on CMA, pseudohyphae and hyphae are not formed. Ascospores formation is not observed in any of the strains or when strains are paired on CMA, 5% ME agar, Fowell’s acetate agar, Gorodkowa agar or V8 agar. Fermentation is absent. The following carbon compounds are assimilated in liquid medium: glucose, inulin, sucrose, trehalose (weak), maltose (delayed), melezitose (weak), methyl-α-D-glucoside (delayed), cellobiose (delayed), salicin (weak), D-xylose (weak), L-arabinose (delayed and weak), D-arabinose, D-ribose (delayed and weak), glycerol, ribitol, D-mannitol (weak), D-glucitol (delayed), and succinate. No growth occurred in raffinose, melibiose, galactose, lactose, L-sorbose, L-rhamnose, 5-keto-D-gluconate, methanol, ethanol, erythritol, galactitol, myo-inositol, DL-lactate, citrate, D-gluconate, D-glucosamine, N-acetyl-D-glucosamine, 2-keto-D-gluconate, D-glucuronate, and glucono-1, 5-lactone. Nitrate, nitrite (weak), and ethylamine (delayed and weak) are utilized as a nitrogen source. L-Lysine and cadaverine are not utilized. Growth in vitamin-free medium is positive. No growth occurred with 1% acetic acid, 0.01% cycloheximide or 10% NaCl plus 5% glucose. Maximum growth temperature is 25 °C. Starch-like polysaccharide is produced. Urease reaction is positive. Diazonium blue B reaction is negative.

##### Additional strain examined.

CHINA • Guizhou Prov.: Pingtang County, Sifangjing Village, on the phylloplane of *Rhododendron simsii*, Mar. 2024, D. Lu, NYNU 243137.

##### GenBank accession numbers.

Holotype GDMCC 2.543^T^ (ITS: PV839639, D1/D2: PV839638, *mtSSU*: PX939035); additional strains NYNU 243137 (ITS: PX070534, D1/D2: PX070535, *mtSSU*: PX939144).

#### Taphrina foliicola

Taxon classificationFungiHaplotaxidaTaphrinaceae

C.Y. Chai & F.L. Hui sp. nov.

EF37AABB-EC93-59C2-A4C3-2CC2D3DFA06C

861669

Fig. 2I, J

##### Etymology.

The specific epithet foliicola refers to the ex-type strain isolated from a leaf.

##### Diagnosis.

Based on multi-gene phylogenetic analysis, *T. foliicola* formed a clade clustered with *T. carpini*, *T. inositophila*, and *T. virginica* with significant support values (Fig. 1). *T. foliicola* differed from those three known species in this clade by more than three nucleotide substitutions (0.5%) in the LSU region and over 35 nucleotide mismatches (5.9%) in the ITS region (Suppl. material 2: table S5). Physiologically, *T. foliicola* can be distinguished from *T. carpini*, *T. inositophila*, and *T. virginica* in its inability to assimilate D-arabinose, ribitol, and citrate (Table 2; Suppl. material 2: table S5).

##### Typus.

CHINA • Henan Prov.: Neixiang County, Baotianman Nature Reserve, on the phylloplane of *Carpinus turczaninowii*, Sep. 2022, J.Z. Li, NYNU 2211137 (holotype GDMCC 2.330^T^ preserved as a metabolically inactive state, culture ex-type PYCC 9942).

##### Description.

On YM agar after 7 days at 20 °C, the streak culture is yellowish-cream, butyrous, and smooth, with an entire margin. In YM broth after 3 days at 20 °C, the cells are ellipsoidal and cylindrical, 3.1–3.9 × 5.6–8.2 μm, occurring singly or with buds. After 1 month at 20 °C, a ring and a sediment are present. In Dalmau plate culture on CMA, pseudohyphae and hyphae are not formed. Ascospores formation is not observed in any of the strains or when strains are paired on CMA, 5% ME agar, Fowell’s acetate agar, Gorodkowa agar or V8 agar. Fermentation is absent. The following carbon compounds are assimilated in liquid medium: glucose, inulin (delayed), sucrose, raffinose (delayed and weak), trehalose, maltose, melezitose, cellobiose, salicin (delayed and weak), D-xylose, glycerol, D-mannitol, D-glucitol, succinate (delayed), and N-acetyl-D-glucosamine. No growth occurred in melibiose, galactose, lactose, methyl-α-D-glucoside, L-sorbose, L-rhamnose, L-arabinose, D-arabinose, 5-keto-D-gluconate, D-ribose, methanol, ethanol, erythritol, ribitol, galactitol, myo-inositol, DL-lactate, citrate, D-gluconate, D-glucosamine, 2-keto-D-gluconate, D-glucuronate, and glucono-1, 5-lactone. Nitrite, nitrate (delayed), and L-lysine are utilized as a nitrogen source. Ethylamine and cadaverine are not utilized. Growth in vitamin-free medium is positive. No growth occurred with 1% acetic acid, 0.01% cycloheximide or 10% NaCl plus 5% glucose. Maximum growth temperature is 25 °C. Starch-like polysaccharide is produced. Urease reaction is positive. Diazonium blue B reaction is negative.

##### Additional strain examined.

CHINA • Henan Prov.: Neixiang County, Baotianman Nature Reserve, on the phylloplane of *Carpinus turczaninowii*, Sep. 2022, J.Z. Li, NYNU 221115; Henan Prov.: Songxian County, Tianchi mountain, on the phylloplane of *Carpinus turczaninowii*, Jun. 2023, J.Z. Li, NYNU 236258; Henan Prov.: Songxian County, Tianchi mountain, on the phylloplane of *Cornus officinalis*, Jun. 2023, J.Z. Li, NYNU 236334.

##### GenBank accession numbers.

Holotype GDMCC 2.330^T^ (ITS: OP959653, D1/D2: OP959502, *mtSSU*: PX939147); additional strains NYNU 221115 (ITS: OP954718, D1/D2: OP954686, *mtSSU*: PX939038), NYNU 236258 (ITS: PX063794, D1/D2: PX070529, *mtSSU*: PX939042), and NYNU 236334 (ITS: PX063790, D1/D2: PX070528, *mtSSU*: PX939043).

##### Notes.

An extra Chinese strain CGMCC 2.2537 had identical LSU sequences and a similar ITS sequence (with two nucleotide difference) with *T. foliicola*, which indicated that this new species has a wide geographical distribution.

#### Srisukozyma

Taxon classificationFungi

C.Y. Chai & F.L. Hui gen. nov.

C4E8426A-A58E-5F57-84E0-451C9D4A3B83

861671

##### Type species.

*Srisukozyma musae*.

##### Etymology.

The genus is named in honor of Nantana Srisuk for his contributions to yeast taxonomy.

##### Description.

Colonies are yellowish-cream and butyrous. Budding cells are present, and yeast cells divide by multilateral budding. Neither pseudohyphae nor hyphae are produced. Ascospores have not been observed in either individual or mixed cultures. Glucose is not fermented. Diazonium Blue B reaction and urease activity are negative.

##### Classification.

*Taphrinales*, *Taphrinomycetes*, *Taphrinomycotina*, *Ascomycota*.

##### Notes.

The new genus *Srisukozyma* is proposed for the branch represented by two groups NYNU 24437 and NYNU 2411186, which was closely related to *Savitreella*. The genus is mainly circumscribed by the phylogenetic analysis of the combined ITS, LSU, and *mtSSU* dataset, in which it occurred as a separate branch within the *Taphrinales*. The genus *Srisukozyma* can also be differentiated from its closely related genus *Savitreella* based on physiological characteristics (Table 2). *Srisukozyma* species do not assimilate cellobiose, or D-gluconate, whereas species of *Savitreella* are capable of utilizing these substrates. Moreover, *Srisukozyma* species are able to assimilate raffinose, a trait not shared by species of *Savitreella*.

#### Srisukozyma musae

Taxon classificationFungi

C.Y. Chai & F.L. Hui sp. nov.

91DA7889-C9F2-5FD7-828A-04AB06FE9A6D

861672

Fig. 3A, B

##### Etymology.

The specific epithet musae refers to *Musa*, the plant genus from which the type strain was isolated.

**Figure 3.**
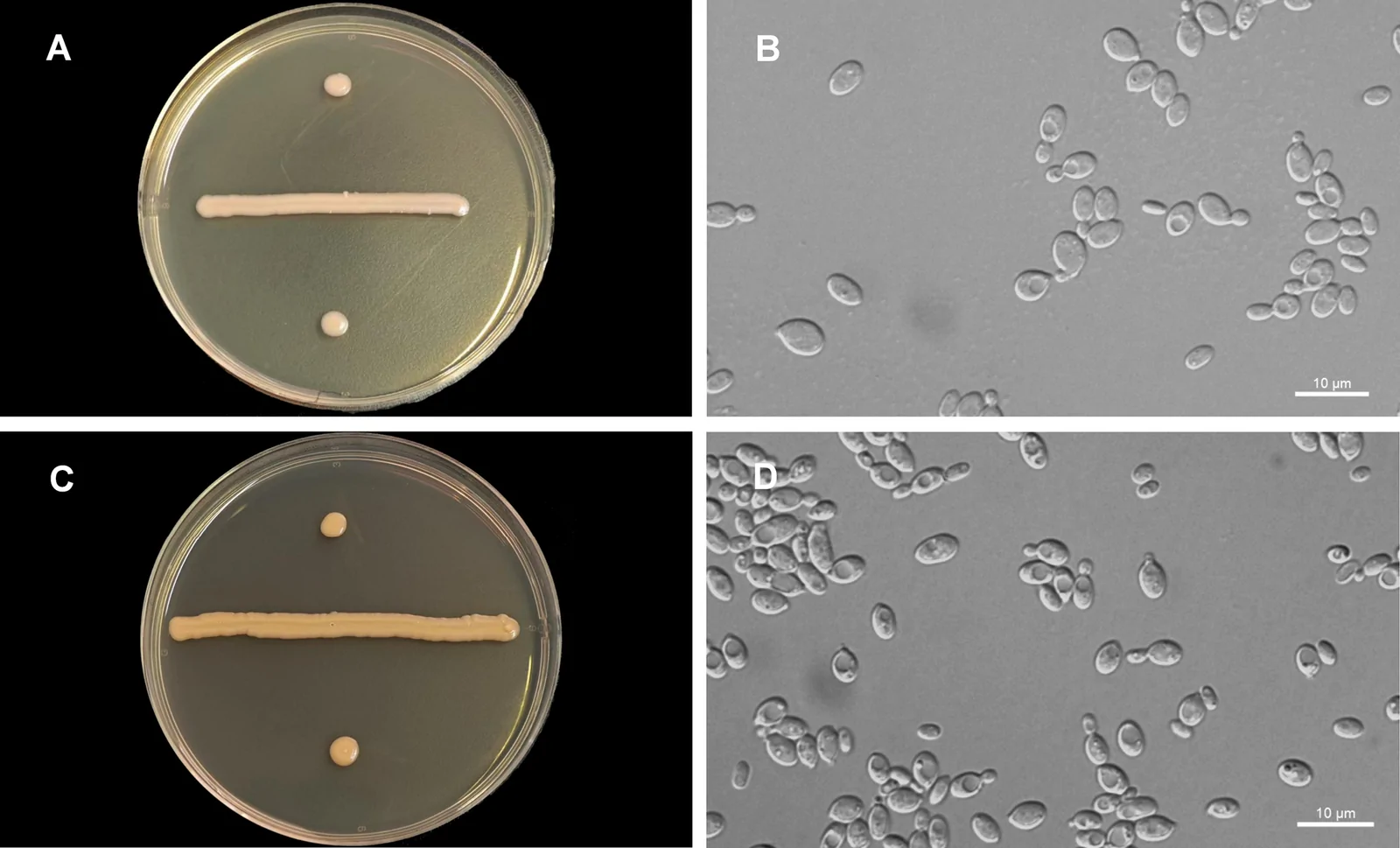
The streak culture grown on YM agar for 7 d at 20 °C and vegetative cells grown in YM broth for 3 d at 20 °C. **A, B**. *Sr. musae* NYNU 24437. **C, D**. *Sr. ovata* NYNU 2411186. Scale bars: 10 μm.

##### Diagnosis.

Multi-gene phylogenetic analysis revealed that *Sr. musae* was closely related to *Sr. ovata* (Fig. 1). *Sr. musae* differed from the closely related species *Sr. ovata* by 33 nucleotide substitutions (5.7%) in the LSU region and by 88 nucleotide mismatches (13.9%) in the ITS region (Suppl. material 2: table S6). Physiologically, *Sr. musae* can be differentiated from its closely related species *Sr. ovata* in its inability to assimilate inulin, methyl-α-D-glucoside, L-arabinose, D-arabinose, and citrate (Table 2; Suppl. material 2: table S6).

##### Typus.

CHINA• Hainan Prov.: Sanya City, Madan Village, on the phylloplane of *Musa nana*, Mar. 2024, S.L. Lv, NYNU 24437 (holotype CICC 33645^T^ preserved as a metabolically inactive state, culture ex-type PYCC 10061).

##### Description.

On YM agar after 7 days at 20 °C, the streak culture is yellowish-cream, butyrous, and smooth, with an entire margin. In YM broth after 3 days at 20 °C, the cells are ovoid and ellipsoidal, 2.9–4.3 × 4.0–9.2 μm, occurring singly or with buds. After 1 month at 20 °C, a ring and a sediment are present. In Dalmau plate culture on CMA, pseudohyphae and hyphae are not formed. Ascospores formation is not observed in any of the strains or when strains are paired on CMA, 5% ME agar, Fowell’s acetate agar, Gorodkowa agar or V8 agar. Fermentation is absent. The following carbon compounds are assimilated in liquid medium: glucose, sucrose, raffinose, trehalose, maltose (delayed and weak), melezitose, salicin (delayed and weak), L-sorbose (delayed), D-xylose, ethanol (weak), glycerol, ribitol (delayed and weak), D-mannitol, D-glucitol (delayed and weak), DL-lactate, and succinate. No growth occurred in inulin, melibiose, galactose, lactose, methyl-α-D-glucoside, cellobiose, L-rhamnose, L-arabinose, D-arabinose, 5-keto-D-gluconate, D-ribose, methanol, erythritol, galactitol, myo-inositol, citrate, D-gluconate, D-glucosamine, N-acetyl-D-glucosamine, 2-keto-D-gluconate, D-glucuronate, and glucono-1,5-lactone. Nitrite (delayed and weak), nitrate (delayed and weak), and L-lysine (delayed) are utilized as a nitrogen source. Ethylamine and cadaverine are not utilized. Growth in vitamin-free medium is positive. No growth occurred with 0.01% cycloheximide or 10% NaCl plus 5% glucose. Maximum growth temperature is 30 °C. Diazonium Blue B reaction and urease activity are negative.

##### Additional strain examined.

CHINA• Hainan Prov.: Sanya City, Madan Village, on the phylloplane of *Musa nana*, Mar. 2024, S.L. Lv, NYNU 244126; Hainan Prov.: Sanya City, Baogu Village, on the phylloplane of *Musa nana*, Mar. 2024, S.L. Lv, NYNU 244111; Hainan Prov.: Sanya City, Lanji Village, on the phylloplane of *Musa nana*, Mar. 2024, S.L. Lv, NYNU 24449.

##### GenBank accession numbers.

Holotype CICC 33645^T^ (ITS: PP849688, D1/D2: PP837701, *mtSSU*: PX939036); additional strains NYNU 244126 (ITS: PX070522, D1/D2: PX070523, *mtSSU*: PX939145), NYNU 244111 (ITS: PX070525, D1/D2: PX070524, *mtSSU*: PX939143), and NYNU 24449 (ITS: PX070527, D1/D2: PX070526, *mtSSU*: PX939037).

#### Srisukozyma ovata

Taxon classificationFungi

C.Y. Chai & F.L. Hui sp. nov.

E666146C-7F76-5205-987D-7B8575B78DC8

861674

Fig. 3C, D

##### Etymology.

The specific epithet ovata refers to the ovoid cell morphology of the type strain.

##### Diagnosis.

In the multi-gene phylogenetic tree, *Sr. ovata* clustered with *Sr. musae* with high support values (Fig. 1). *Sr. ovata* differed from the closely related species *Sr. musae* by 33 nucleotide substitutions (5.7%) in the LSU region and by 88 nucleotide mismatches (13.9%) in the ITS region (Suppl. material 2: table S7). Physiologically, *Sr. ovata* can be differentiated from the closely related species *Sr. musae* in its ability to assimilate inulin, methyl-α-D-glucoside, L-arabinose, D-arabinose, and citrate and its inability to assimilate L-sorbose (Table 2; Suppl. material 2: table S7).

##### Typus.

CHINA• Guizhou Prov.: Guiyang City, Guiyang Botanical Garden, on the phylloplane of *Polygonum chinense*, Oct. 2024, D. Lu, NYNU 2411186 (holotype GDMCC 2.542^T^ preserved as a metabolically inactive state, culture ex-type JCM 38267).

##### Description.

On YM agar after 7 days at 20 °C, the streak culture is orange-yellow, butyrous, and smooth, with an entire margin. In YM broth after 3 days at 20 °C, the cells are ovoid and ellipsoidal, 2.3–2.8 × 3.3–6.4 μm, occurring singly or with buds. After 1 month at 20 °C, a pellicle and a sediment are present. In Dalmau plate culture on CMA, pseudohyphae and hyphae are not formed. Ascospores formation is not observed in any of the strains or when strains are paired on CMA, 5% ME agar, Fowell’s acetate agar, Gorodkowa agar or V8 agar. Fermentation is absent. The following carbon compounds are assimilated in liquid medium: glucose, inulin, sucrose, raffinose (delayed), trehalose, maltose, melezitose, methyl-α-D-glucoside (delayed and weak), salicin (delayed and weak), D-xylose (delayed), L-arabinose (delayed and weak), D-arabinose (delayed and weak), ethanol, glycerol, ribitol (delayed and weak), D-mannitol, D-glucitol (delayed), DL-lactate (delayed and weak), succinate, and citrate. No growth occurred in melibiose, galactose, lactose, cellobiose, L-sorbose, L-rhamnose, 5-keto-D-gluconate, D-ribose, methanol, erythritol, galactitol, myo-inositol, D-gluconate, D-glucosamine, 2-keto-D-gluconate, D-glucuronate, and glucono-1, 5-lactone. Nitrite (delayed and weak), nitrate (delayed and weak), ethylamine (delayed and weak), and L-lysine are utilized as a nitrogen source. Cadaverine is not utilized. Growth in vitamin-free medium is positive. No growth occurred with 0.01% cycloheximide or 10% NaCl. Maximum growth temperature is 30 °C. Starch-like polysaccharide is produced. Diazonium Blue B reaction and urease activity are negative.

##### Additional strain examined.

CHINA • Guizhou Prov.: Guiyang City, Guiyang Botanical Garden, on the phylloplane of *Polygonum chinense*, Oct. 2024, D. Lu, NYNU 2411221.

##### GenBank accession numbers.

Holotype GDMCC 2.542^T^ (ITS: PV843161, D1/D2: PV839685, *mtSSU*: PX939146); additional strain NYNU 2411221 (ITS: PX070537, D1/D2: PX070536, *mtSSU*: PX939149).

## Discussion

Species of the genus *Taphrina* have traditionally been recognized based on their host range, geographical distribution, morphology of sexual forms, and their effects on host plant tissues (Mix 1949; Petrýdesová et al. 2013, 2016). These species are also distinguished by a unique combination of physiological characteristics and the carbohydrate composition of their cell walls (Bacigálová et al. 2003; Petrýdesová et al. 2016). However, *Taphrina* may not be strictly associated with specific host plants (Mix 1949; Fonseca and Rodrigues 2011), and some species without sexual states lack clear associations with particular host plants (Inácio et al. 2004; Petrýdesová et al. 2016). The application of molecular tools for species identification has facilitated the connection between sexual and asexual morphs, even when the two forms are morphologically dissimilar (Selbmann et al. 2014). Following the polyphasic approach recommended by Selbmann et al. (2014) and Petrýdesová et al. (2016), the present study identified five new *Taphrina* species and one new genus *Srisukozyma* with two new species, all of which were observed only in their yeast states.

To date, 43 species are phylogenetically recognized in *Taphrina*, including *T. aspidistrae*, *T. cunninghamiae*, *T. foliicola*, *T. fujianensis*, and *T. guizhouensis* from the present study (Petrýdesová et al. 2016). Among the species of the genus *Taphrina*, at least four, *T. caerulescens*, *T. populina*, *T. robinsoniana*, *T. wiesneri*, exhibit high intraspecific sequence variation (Rodrigues and Fonseca 2003). For example, three strains of *T. populina* had similar LSU sequences (with a three nucleotides difference), but possessed 13–14 nucleotide differences (2.1–2.2%) in the ITS region. In the case of *T. wiesneri*, four strains belong to this species differed from each other by one nucleotide substitutions in the LSU region and 1–14 nucleotide mismatches (0.2–2.3%) in the ITS region. The result of sequence similarity analysis indicated that members of the genus *Taphrina* exhibit high intraspecific sequence variation, but high intraspecific sequence variation was found only in the ITS region, not in the LSU region (Rodrigues and Fonseca 2003). In agreement with previous observation, intraspecific variability was also reported for mating strains (CBS 490 and CBS 2630) of the heterothallic species *Sporidiobolus salmonicolor*, which were found to differ from one another by seven nucleotide substitutions (1.2%) and 26 nucleotide mismatches (4.5%) in the LSU and ITS regions, respectively (Dayo-Owoyemi et al. 2013). Although the ITS region was selected as a universal DNA-barcode for fungal classification, primarily due to its high discriminative power (Schoch et al. 2012), it is clear from this study that this region alone is not sufficient for species delimitation in yeast species, especially within the *Taphrina* clade. Thus, it is important to keep in mind that both gene regions are necessary for a reliable species delimitation in the *Taphrina* clade.

Two new *Srisukozyma* species clustered in the clade as *Savitreella* with strong support. However, low sequence similarity was observed between *Srisukozyma* species and any known taxa. The phylogenetic analysis and the comparison of predicted taxonomic thresholds demonstrated that *Srisukozyma* represents a new genus within the *Taphrinales*. The new genus is also physiologically distinct from other genera within *Taphrinales*. For instance, *Srisukozyma* species do not assimilate cellobiose, whereas species of the genera *Savitreella* and *Taphrina* are capable of utilizing these substrates. Moreover, *Srisukozyma* species are able to assimilate raffinose, a trait not shared by species of *Savitreella*. Since the family assignment of the *Taphrinomycotina* is still unclear, we chose not to assign the family of the new genus and have left open the family assignment until the existing family structure within the *Taphrinomycotina* is better clarified, based on eventual phylogenomic data. Although the three-locus dataset (ITS, LSU, *mtSSU*) used in this study has inherent limitations, this marker combination is the current standard for species-level taxonomy in *Taphrinales*. Together with extensive physiological and morphological data, it provides robust support for the new taxa described herein. Future phylogenomic analyses based on whole-genome sequencing, even at low coverage, will be necessary to resolve deeper nodes within *Taphrinomycotina* and to refine family-level classifications.

Members of *Taphrina* are widely distributed and inhabit various environments. The sexual, filamentous state exclusively exists as a biotrophic plant pathogen on *Rosaceae*, *Betulaceae*, and *Fagaceae* (Selbmann et al. 2014). The asexual yeast states exhibit saprobic growth and have been isolated from a variety of substrates, including flowers (Golonka and Vilgalys 2013), leaves (Inácio et al. 2004; Petrýdesová et al. 2016), leaf litter (Babjeva and Reshetova 1998; Maksimova and Chernov 2004; Yurkov et al. 2004), forest soil (Wuczkowski et al. 2005), and even Antarctic rock (Selbmann et al. 2014). Furthermore, ITS sequences matching various *Taphrina* species were identified among endophytic fungi colonizing bur oak (*Quercus macrocarpa*) in the Flint Hills region, USA (Jumpponen and Jones 2009, 2010), and beech (*Fagus sylvatica*) in the French Pyrenees (Cordier et al. 2012). Among all the reported *Taphrina* species, most are associated with plant leaves. The five new species described in this paper were also isolated from leaf surfaces, further enriching the diversity of phylloplane fungi. Additionally, members of *Taphrina* are generally recognized as phytopathogens. Many *Taphrina* species are plant parasites that cause gall formation on stems, leaf curls, and witches’ brooms, particularly on economically important fruit trees such as peach, plum, and cherry (Selbmann et al. 2014). Consequently, the growing interest in these fungi extends beyond their taxonomy and ecological roles to include their potential pathogenicity.

## Conclusion

This study reveals a new monotypic genus *Srisukozyma*, comprising two new species *Sr. musae* and *Sr. ovata*, along with five additional new *Taphrina* species: *T. aspidistrae*, *T. cunninghamiae*, *T. foliicola*, *T. fujianensis*, and *T. guizhouensis*, all isolated from leaf surfaces at different locations across China. Phylogenetic analyses and phenotypic characterization support the seven new taxa as members of *Taphrinales* (Fig. 1). Detailed descriptions, illustrations, and comparative discussions with their nearest relatives are provided. This study represents the first comprehensive analysis of *Taphrinales* species associated with plant materials from tropical and subtropical China. The findings expand our understanding of generic and species diversity within this order. We anticipate that future research will uncover more species and genera within the order.

## Supplementary Material

XML Treatment for Taphrina aspidistrae

XML Treatment for Taphrina cunninghamiae

XML Treatment for Taphrina fujianensis

XML Treatment for Taphrina guizhouensis

XML Treatment for Taphrina foliicola

XML Treatment for Srisukozyma

XML Treatment for Srisukozyma musae

XML Treatment for Srisukozyma ovata

## Additional information

### Artificial Intelligence (AI) use

The authors accept full responsibility for the content of the manuscript, including the disclosure of any use of AI.

No AI tools were used in the preparation of this manuscript.

### Adherence to national and international regulations

All the fungal strains used in this study have been legally obtained, respecting the Convention on Biological Diversity (Rio Convention).

### Author ORCIDs

C.-Y. Chai https://orcid.org/0000-0001-7753-6223

D.-Y. Cai https://orcid.org/0009-0002-6866-7322

B.-Y. Song https://orcid.org/0009-0000-6802-5077

R.-X. Wang https://orcid.org/0009-0009-5141-7096

F.-L. Hui https://orcid.org/0000-0001-7928-3055

### Data availability

All gene sequences generated in this study are available in GenBank under the accession numbers provided, with all additional data included in the manuscript and its supplements.

## Supplementary materials

Supplementary material 1 A dataset of the combined ITS, LSU, and *mtSSU* sequences for Fig. 1 Data type fas Explanation note The concatenated dataset of the three regions had a total length of 1,979 bp, consisting of 598 bp for the ITS region, 574 bp for the LSU region, and 807 bp for the *mtSSU* region. https://binary.pensoft.net/file/1753480This dataset is made available under the Open Database License (http://opendatacommons.org/licenses/odbl/1.0/). The Open Database License (ODbL) is a license agreement intended to allow users to freely share, modify, and use this Dataset while maintaining this same freedom for others, provided that the original source and author(s) are credited. Chun-Yue Chai, Dan-Yang Cai, Bing-Yan Song, Rui-Xiu Wang, Feng-Li Hui

Supplementary material 2 Supplementary table SS1–S7 Data type xlsx Explanation note Physiological characteristics and nucleotide divergences that differentiate new species from their closest species. Each new species provided as a separate Excel sheet. https://binary.pensoft.net/file/1753481This dataset is made available under the Open Database License (http://opendatacommons.org/licenses/odbl/1.0/). The Open Database License (ODbL) is a license agreement intended to allow users to freely share, modify, and use this Dataset while maintaining this same freedom for others, provided that the original source and author(s) are credited. Chun-Yue Chai, Dan-Yang Cai, Bing-Yan Song, Rui-Xiu Wang, Feng-Li Hui
